# Longitudinal Change of Clinical and Biological Measures in Early Parkinson's Disease: Parkinson's Progression Markers Initiative Cohort

**DOI:** 10.1002/mds.27361

**Published:** 2018-03-23

**Authors:** Tanya Simuni, Andrew Siderowf, Shirley Lasch, Chris S. Coffey, Chelsea Caspell‐Garcia, Danna Jennings, Caroline M. Tanner, John Q. Trojanowski, Leslie M. Shaw, John Seibyl, Norbert Schuff, Andrew Singleton, Karl Kieburtz, Arthur W. Toga, Brit Mollenhauer, Doug Galasko, Lana M. Chahine, Daniel Weintraub, Tatiana Foroud, Duygu Tosun, Kathleen Poston, Vanessa Arnedo, Mark Frasier, Todd Sherer, Sohini Chowdhury, Kenneth Marek, the Parkinson's Progression Marker Initiative*

**Affiliations:** ^1^ Northwestern University Chicago Illinois USA; ^2^ University of Pennsylvania Philadelphia Pennsylvania USA; ^3^ Institute for Neurodegenerative Disorders New Haven Connecticut USA; ^4^ University of Iowa Iowa City Iowa USA; ^5^ Eli Lilly Indianapolis Indiana USA; ^6^ University of California San Francisco California USA; ^7^ National Institute on Aging National Institutes of Health Bethesda Maryland USA; ^8^ Clinical Trials Coordination Center University of Rochester Rochester New York USA; ^9^ University of Southern California Los Angeles California USA; ^10^ Paracelsus‐Elena Klinik Kassel Germany; ^11^ University of California San Diego California USA; ^12^ Indiana University Indianapolis Indiana USA; ^13^ Stanford University Medical Center Stanford California USA; ^14^ Michael J Fox Foundation New York NY USA

**Keywords:** Parkinson's disease, disease subtypes, tremor dominant, postural instability, gait disorder predominant

## Abstract

**Objective:** The objective of this study was to assess longitudinal change in clinical and dopamine transporter imaging outcomes in early, untreated PD.

**Methods:** We describe 5‐year longitudinal change of the MDS‐UPDRS and other clinical measures using results from the Parkinson's Progression Markers Initiative, a longitudinal cohort study of early Parkinson's disease (PD) participants untreated at baseline. We also provide data on the longitudinal change in dopamine transporter 123‐I Ioflupane striatal binding and correlation between the 2 measures.

**Results:** A total of 423 PD participants were recruited, and 358 remain in the study at year 5. Baseline MDS‐UPDRS total score was 32.4 (standard deviation 13.1), and the average annual change (assessed medications OFF for the treated participants) was 7.45 (11.6), 3.11 (11.7), 4(11.9), 4.7 (11.1), and 1.74(11.9) for years 1, 2, 3, 4, and 5, respectively (*P* < .0001 for the change over time), with a steeper change in year 1. Dopaminergic therapy had a significant effect on the change of MDS‐UPDRS. There was a significant longitudinal change in dopamine transporter binding in all striatal regions (*P* < .001). There was a significant but weak correlation between MDS‐UPDRS and dopamine transporter binding at baseline and years 1, 2, and 4, but no correlation between the rate of change of the 2 variables.

**Conclusions:** We present 5‐year longitudinal data on the change of the MDS‐UPDRS and other clinical and dopamine transporter imaging outcome measures in early PD. These data can be used for sample size estimates for interventional studies in the de novo PD population. © 2018 The Authors. Movement Disorders published by Wiley Periodicals, Inc. on behalf of International Parkinson and Movement Disorder Society.

Parkinson's disease (PD) is the second most common neurodegenerative disease. Although there is a large armamentarium of effective symptomatic therapies, disease‐modifying interventions are an area of tremendous unmet need. One of the limitations in the development of therapeutics for PD disease modification is the lack of reliable, objective measures of PD progression. In the absence of objective measures, disease modification trials have traditionally recruited PD participants de novo at baseline and use either change in the Unified Parkinson's Disease Rating Scale (UPDRS) 1 or time to initiation of symptomatic therapy as the primary outcome measures.^2^, ^3^ The Parkinson's Progression Markers Initiative (PPMI) is an ongoing observational, international, multicenter cohort study aimed to identify the clinical, serological, genetic, cerebrospinal fluid (CSF) and imaging biomarkers of PD progression in a large cohort of participants including de novo PD patients and healthy controls. PPMI participants are assessed every 6 months with a spectrum of clinical measures, including the MDS‐UPDRS and an annual collection of biological and imaging data.

In 2001, the Movement Disorder Society (MDS) convened a task force to develop a new version of UPDRS.^4^ The MDS Unified Parkinson's Disease Rating Scale (MDS‐UPDRS) underwent extensive clinimetric development and was endorsed by the MDS as the preferred tool to measure PD disability.^5^ There are limited published data on the longitudinal rate of change of the scale in the de novo PD population. Such data are important to understand how the MDS‐UPDRS may perform when used as an outcome measure in interventional clinical trials conducted in patients with early PD. Dopamine transporter (DAT) 123‐I Ioflupane (DatScan) single‐photon emission computed tomography imaging is the only commercially approved functional imaging modality to establish presence of presynaptic dopamine deficiency. In clinical practice, DAT imaging is interpreted qualitatively based on the visual interpretation, whereas quantitative analysis is routinely used in the research domain. The scan is increasingly used in clinical trials to exclude patients without evidence of dopamine deficiency who are unlikely to have the pathology that typically causes PD.^6^ There are limited data on the sensitivity of DAT binding to longitudinal change, which is an essential question if DAT imaging is to be used as an imaging biomarker in PD clinical trials.

The analyses in this report has 2 main aims: first, to describe the 5‐year change of the MDS‐UPDRS, other clinical outcome measures, and DAT binding; and second to assess the correlation between MDS‐UPDRS and DAT binding in this early PD cohort.

## Methods

Newly diagnosed, de novo PD patients (N = 423) were enrolled in PPMI. At baseline, the PD participants were required to (1) have a recent idiopathic PD diagnosis, (2) be untreated for PD, (3) have DAT deficit, and (4) not have dementia as determined by the site investigator. The aims, methodology, and scope of activities of the study have been previously published.^7^ The study was approved by the institutional review boards at each site, and the participants provided written informed consent. The dataset was downloaded on October 23, 2017. Two key outcome measures were examined. First, MDS‐UPDRS is assessed at every study visit. Once participants start dopaminergic therapy (DT), defined as levodopa and/or dopamine agonists, the MDS‐UPDRS is assessed in the OFF medications state defined in the PPMI protocol as more than 6 hours post–last dose of DT and ON state(approximately an hour after the last dose of DT). Participants treated with other PD medications (non‐DT; monoamine oxidase inhibitors and/or anticholinergics and amantadine) are examined only in the ON state. Once participants start any type of DT, the dose is reported as cumulative levodopa equivalence daily dose as well as levodopa equivalence daily dose by DT subclass.^8^ Second, all participants underwent DAT imaging at baseline and years 1, 2, and 4. Imaging results are analyzed according to the imaging technical operations manual (http://ppmi-info.org/). DAT imaging data are presented as both the regional specific binding ratio and percent change of the specific binding ratio by striatal subregion. Ipsilateral versus contralateral are defined in relation to the more clinically affected body side at baseline. PPMI dataset also includes CSF measures of the following putative PD biomarkers: β‐amyloid 1‐42, total tau, tau phosphorylated at threonine 181, and unphosphorylated α‐synuclein. CSF measures are not included in this analysis because the 1‐year longitudinal data have been recently reported,^9^ and 3‐year data will be reported shortly.

### Statistical Analysis

Summary statistics for baseline demographics and PD characteristics were reported for all PD patients. Repeated‐measures linear mixed models were used to examine the changes in MDS‐UPDRS total and part III scores, separately for the whole cohort, and for the following subsets of participants: (1) untreated, (2) treated with DT (defined as levodopa and/or dopamine agonists), (3) treated only with levodopa, and (4) treated with other PD medications. Repeated‐measures linear mixed models were also used to examine the changes in clinical characteristics and DAT binding over time for the whole cohort.

Spearman correlations were calculated between MDS‐UPDRS and DAT binding ratios at each time point for the whole cohort and for the subset treated only with levodopa (using OFF scores in treated patients) and also between the change in MDS‐UPDRS and percent change in DAT binding ratios for the whole cohort. The *t*‐tests were used in pairwise comparisons of the 1‐year change in MDS‐UPDRS total score for patients who were untreated, treated with DT, and treated with other PD medications at year 1.

## Results

**Figure 1:**
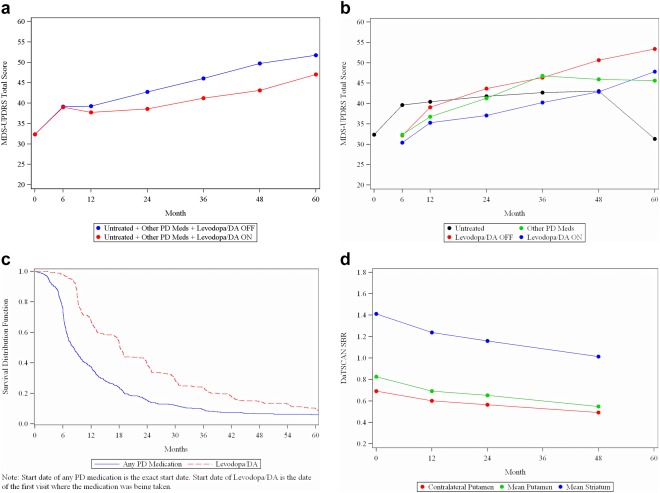
(a) MDS‐UPDRS Total Score over time in all PD subjects. (b) MDS‐UPDRS Total Score over time in PD subjects by treatment group. (c) Kaplan‐Meier curve for time to start PD medications in PD subjects. (d) DaTSCAN over time in PD subjects.

Baseline demographics and disease characteristics for the 423 PD participants are presented in Table 1 and discussed in the companion paper. The participants' demographics are generally consistent with early PD clinical trials populations. The data on 5‐year study retention are presented in Table 2. At the time of data download, 85% of participants remained in the study. The 5‐year longitudinal data on MDS‐UPDRS are presented in Table 2 and Figure 1A,B. The data are presented for the overall cohort and by treatment status as discussed in the Methods section. The numbers reflect all patients who were seen at that time point and who had data on MDS‐UPDRS available. Smaller datasets for 36 months and beyond reflect the fact that PPMI is an ongoing study and data continue to be collected. Discrepancies between the number of patients seen and the number included in the MDS‐UPDRS reporting reflect missing data largely driven by incomplete data collection predominantly in the medications OFF state. Consistent with the previously published studies, 59% of the PPMI cohort started any PD medications by year 1 and 42% started DT (Fig. 1C).^10^ MDS‐UPDRS data for the PPMI participants are presented for the following groups: (1) untreated + treated OFF, which includes participants on no medication and on non‐DT PD medications and participants on DT examined in the medications OFF state; (2) untreated + treated ON, which includes participants on no medication, participants on non‐DT PD medications, and participants on DT evaluated in the ON state as well as the subgroups of (1) untreated, (2) treated with DT both OFF and ON, (3) treated only with levodopa both OFF and ON, and (4) treated with non‐DT PD medications (Table 2).

**Table 1:** Baseline demographics and PD characteristics

Variable	PD patients, N = 423
Age	
Mean (SD)	61.66 (9.7)
(Min, max)	(33.5, 84.9)
Missing	0
Age, n (%)	
<56 years	116 (27.42)
56‐65 years	151 (35.70)
>65 years	156 (36.88)
Missing	0
Gender, n (%)	
Male	277 (65.48)
Female	146 (34.52)
Missing	0
Education, n (%)	
<13 years	76 (17.97)
13‐23 years	344 (81.32)
>23 years	3 (0.71)
Missing	0
Ethnicity, n (%)	
Hispanic/Latino	9 (2.13)
Not Hispanic/Latino	414 (97.87)
Missing	0
Race, n (%)	
White	391 (92.43)
Black/African American	6 (1.42)
Asian	8 (1.89)
Other	18 (4.26)
Missing	0
Family history of PD, n (%)^a^	
Any family members with PD	103 (24.41)
No family members with PD	319 (75.59)
Missing	1
Disease duration, mo	
Mean (SD)	6.71 (6.6)
(Min, max)	(0.4, 35.8)
Missing	0
Age of PD onset	
Mean (SD)	59.65 (10.0)
(Min, max)	(25.4, 83.0)
Missing	0
Side most affected, n (%)	
Left	179 (42.32)
Right	234 (55.32)
Symmetric	10 (2.36)
Missing	0
MDS‐UPDRS mean (SD) score & subscores	
MDS‐UDPRS total score	32.36 (13.1)
MDS‐UDPRS part I score	5.57 (4.1)
MDS‐UDPRS part II score	5.90 (4.2)
MDS‐UDPRS part III score	20.89 (8.9)
Missing	1
Hoehn & Yahr, n (%)	
Stage 0	0 (0)
Stage 1	185 (43.74)
Stage 2	236 (55.79)
Stage 3‐5	2 (0.47)
Missing	0
Modified Schwab & England ADL	
Mean (SD)	93.14 (5.9)
(Min, max)	(70.0, 100.0)
Missing	0
TD/non‐TD classification, n (%)	
TD	299 (70.85)
Non‐TD	123 (29.15)
Missing	1
PIGD score	
Mean (SD)	0.23 (0.2)
(Min, max)	(0.0, 1.4)
Missing	1
Tremor score	
Mean (SD)	0.49 (0.3)
(Min, Max)	(0.0, 1.8)
Missing	1
MOCA	
Mean (SD)	27.13 (2.3)
(Min, max)	(17.0, 30.0)
Missing	3

Report generated on data submitted as of October 23, 2017.

a Family history captures any family member and not restricted to the first‐degree relatives.

TD, tremor dominant; PIGD, postural instability gait disorder predominant.

**Table 2:** MDS‐UPDRS total scores over time in treated and untreated PD patients

	Baseline	Month 6	Month 12	Month 24	Month 36	Month 48	Month 60	
Variable	n expected/n seen at visit	n expected/n seen at visit	n expected/n seen at visit	n expected/n seen at visit	n expected/n seen at visit	n expected/n seen at visit	n expected/n seen at visit	*P* value, change over time
Untreated + Treated OFF, n	423/423	414/402	n = 409/394	399/376	388/365	377/335	240/218	
Total score								<.0001
n completed	422	400	334	282	255	249	163	
Mean (SD)	32.36 (13.1)	39.16 (16.0)	39.28 (16.4)	42.75 (17.0)	46.03 (18.6)	49.70 (20.1)	51.77 (20.3)	
(Min, max)	(7.0, 72.0)	(4.0, 94.0)	(5.0, 113.0)	(10.0, 99.0)	(9.0, 121.0)	(9.0, 128.0)	(16.0, 140.0)	
Part III score								<.0001
n completed	423	400	334	282	255	249	163	
Mean (SD)	20.89 (8.9)	24.87 (10.3)	25.10 (11.1)	27.13 (11.4)	29.22 (12.2)	31.53 (12.3)	32.15 (12.8)	
(Min, max)	(4.0, 51.0)	(3.0, 60.0)	(2.0, 67.0)	(3.0, 68.0)	(4.0, 80.0)	(6.0, 80.0)	(6.0, 90.0)	
Untreated + Treated ON								
Total score								<.0001
n completed	422	402	382	354	343	321	204	
Mean (SD)	32.36 (13.1)	39.04 (16.0)	37.74 (16.1)	38.55 (16.3)	41.25 (18.6)	43.10 (21.0)	47.03 (22.7)	
(Min, max)	(7.0, 72.0)	(4.0, 94.0)	(4.0, 113.0)	(5.0, 99.0)	(3.0, 118.0)	(6.0, 142.0)	(13.0, 156.0)	
Part III score								<.0001
n completed	423	402	382	354	343	321	204	
Mean (SD)	20.89 (8.9)	24.80 (10.4)	23.43 (10.9)	23.13 (11.4)	24.08 (12.2)	24.31 (13.1)	26.33 (13.3)	
(Min, max)	(4.0, 51.0)	(3.0, 60.0)	(1.0, 67.0)	(0.0, 68.0)	(0.0, 65.0)	(1.0, 70.0)	(3.0, 85.0)	
Untreated, n	423	373	162	58	27	16	9	
Total score								<.0001
n completed	422	373	162	58	27	16	9	
Mean (SD)	32.36 (13.1)	39.66 (15.9)	40.44 (16.3)	41.78 (18.1)	42.70 (20.6)	43.06 (27.3)	31.33 (9.5)	
(Min, max)	(7.0, 72.0)	(4.0, 94.0)	(13.0, 113.0)	(13.0, 99.0)	(11.0, 83.0)	(19.0, 128.0)	(19.0, 48.0)	
Part III score								<.0001
n completed	423	373	162	58	27	16	9	
Mean (SD)	20.89 (8.9)	25.31 (10.2)	26.52 (10.6)	28.09 (12.7)	28.81 (13.2)	28.06 (14.5)	22.67 (6.6)	
(Min, max)	(4.0, 51.0)	(3.0, 60.0)	(6.0, 67.0)	(4.0, 68.0)	(7.0, 52.0)	(10.0, 69.0)	(15.0, 33.0)	
Levodopa/DA OFF	0	20	166	257	296	296	201	
Total score								<.0001
n completed	N/A	18	106	163	186	210	146	
Mean (SD)	N/A	32.17 (17.4)	39.08 (17.5)	43.65 (17.5)	46.34 (19.1)	50.62 (19.9)	53.37 (20.2)	
(Min, max)	N/A	(11.0, 61.0)	(8.0, 89.0)	(10.0, 96.0)	(9.0, 121.0)	(11.0, 111.0)	(17.0, 140.0)	
Part III score								<.0001
n completed	N/A	18	106	163	186	210	146	
Mean (SD)	N/A	18.78 (11.7)	24.31 (11.9)	26.91 (11.5)	28.96 (12.6)	31.77 (12.3)	32.93 (13.0)	
(Min, max)	N/A	(5.0, 51.0)	(2.0, 60.0)	(3.0, 62.0)	(4.0, 80.0)	(6.0, 80.0)	(6.0, 90.0)	
Levodopa/DA ON								
Total score								<.0001
n completed	N/A	20	154	235	274	282	187	
Mean (SD)	N/A	30.40 (17.4)	35.32 (16.1)	37.05 (16.1)	40.26 (18.8)	42.88 (21.1)	47.84 (23.1)	
(Min, max)	N/A	(9.0, 61.0)	(4.0, 83.0)	(5.0, 81.0)	(3.0, 118.0)	(6.0, 142.0)	(13.0, 156.0)	
Part III score								<.0001
n completed	N/A	20	154	235	274	282	187	
Mean (SD)	N/A	17.90 (11.5)	20.40 (10.5)	20.94 (10.8)	22.61 (12.1)	23.49 (13.0)	26.42 (13.6)	
(Min, max)	N/A	(5.0, 51.0)	(1.0, 50.0)	(0.0, 56.0)	(0.0, 65.0)	(1.0, 70.0)	(3.0, 85.0)	
Levodopa only OFF, n	0	11	73	114	124	118	84	
Total score								<.0001
n completed	N/A	10	41	81	81	85	56	
Mean (SD)	N/A	33.00 (16.3)	43.41 (19.5)	45.37 (18.9)	49.26 (20.2)	55.02 (19.8)	58.27 (21.1)	
(Min, max)	N/A	(11.0, 61.0)	(12.0, 89.0)	(10.0, 96.0)	(9.0, 121.0)	(21.0, 111.0)	(17.0, 110.0)	
Part III score								<.0001
n completed	N/A	10	41	81	81	85	56	
Mean (SD)	N/A	18.80 (9.5)	27.37 (12.5)	27.83 (11.5)	31.38 (13.1)	35.55 (12.3)	36.41 (14.4)	
(Min, max)	N/A	(7.0, 38.0)	(9.0, 60.0)	(3.0, 59.0)	(4.0, 80.0)	(8.0, 80.0)	(7.0, 90.0)	
Levodopa only ON								
Total score								<.0001
n completed	N/A	11	73	105	117	116	79	
Mean (SD)	N/A	30.82 (17.1)	37.81 (17.9)	37.77 (17.4)	43.06 (20.4)	46.96 (22.9)	52.23 (26.7)	
(Min, max)	N/A	(9.0, 61.0)	(6.0, 83.0)	(5.0, 80.0)	(7.0, 118.0)	(7.0, 142.0)	(13.0, 156.0)	
Part III score								<.0001
n completed	N/A	11	73	105	117	116	79	
Mean (SD)	N/A	17.55 (9.9)	21.18 (10.7)	20.86 (11.4)	24.15 (12.6)	26.04 (13.9)	28.28 (14.9)	
(Min, max)	N/A	(5.0, 38.0)	(2.0, 50.0)	(0.0, 56.0)	(1.0, 65.0)	(1.0, 70.0)	(3.0, 85.0)	
Other PD meds, n	0	9	66	61	42	23	8	
Total score								<.0001
n completed	N/A	9	66	61	42	23	8	
Mean (SD)	N/A	32.33 (11.8)	36.76 (14.6)	41.26 (14.7)	46.81 (14.5)	45.91 (15.1)	45.63 (18.4)	
(Min, max)	N/A	(13.0, 45.0)	(5.0, 80.0)	(15.0, 74.0)	(13.0, 77.0)	(9.0, 66.0)	(16.0, 75.0)	
Part III score								<.0001
n completed	N/A	9	66	61	42	23	8	
Mean (SD)	N/A	19.00 (9.1)	22.91 (10.4)	26.84 (9.6)	30.67 (9.9)	31.78 (10.6)	28.50 (9.6)	
(Min, max)	N/A	(6.0, 31.0)	(4.0, 47.0)	(6.0, 46.0)	(11.0, 49.0)	(9.0, 48.0)	(9.0, 40.0)	

Different n in OFF and ON scores reflects missing OFF scores where patient either forgot or was unable to withhold medication. Patients are expected at the visit if they are past the expected visit window and have not terminated early from the study.

DA, dopamine agonists; ADLs, activities of daily living; N/A, not applicable.

The annual change of the MDS‐UPDRS in the whole cohort (assessed medications OFF for the treated participants) was 7.45 (SD = 11.6), 3.11 (11.7), 4(11.9), 4.7 (11.1), and 1.74 (11.9) for years 1, 2, 3, 4, and 5, respectively (*P* < .0001 for the change over time). The largest change was in year 1 and plateaued afterward as a reflection of the symptomatic effect of DT. The smallest change at year 5 might reflect a smaller number of patients at that time point. We then calculated the change in MDS‐UPDRS total score from baseline to the year 1 visit (Supporting Information Table 1s). A total of 334 participants had MDS‐UPDRS data at year 1, and data on that subset of participants were used for calculation of the change of the MDS‐UPDRS total score from baseline to year 1 that was 7.5 (SD = 11.6) for the whole cohort. There was a significant increase in MDS‐UPDRS total score over 1 year in all participants. The largest change was in those who remained untreated (10.7 [SD 10.7]), the smallest change was in those who started DT (OFF scores; 2.4 [SD 11.4]), and the group that was treated with non‐DT PD medications fell in between (7.5 [SD 11.2]) the other 2 groups. All pairwise comparisons in change in MDS‐UPDRS total score between groups were significant (Supporting Information Table 1s). Of note, although the PPMI protocol allows OFF assessments to be done ≥6 hours post–last dose of DT, the actual average time to OFF assessment was >12 hours at all time points (Supporting Information Table 2s), and there was no significant effects of the time to ON assessment on the degree of change in DT group (Supporting Information Table 3s). For the participants who started DT, the difference between medications OFF and ON MDS‐UPDRS total score was very modest (3.7 [SD 16.7]) at year 1 (*P* = .07) and increased by year 2 (6.5 [SD 16.7; *P* < .001]) but still remained modest even at year 5. The difference in the MDS‐UPDRS part 3 score in the medication OFF versus ON state was of a similar magnitude (Table 2, Fig. 1a). The levodopa equivalence daily dose totals for specific types of DT at each time point are presented in Supporting Information Table 4s. The analysis of the OFF/ON difference in the levodopa‐only treated group did not change the conclusions.

The 5‐year longitudinal data on the change in the other clinical measures are presented in Table 3. There was a significant change in all measures included, but the magnitude was variable. The change in the MOCA score was small, occurred largely in the first year, and plateaued afterward. MDS‐UPDRS parts I and II scores nearly doubled in 5 years, although the absolute change was still small, in the realm of 5 points for both subscores. The majority of patients remained at the Hoehn and Yahr stage ≤ 2 (93%), which means mild disease, although the average Schwab and England scores dropped from 93.1(SD = 5.9) at baseline to 83.3 (SD = 14.6) at year 5. Detailed analyses of the 2‐ to 3‐year longitudinal change in cognition and other nonmotor symptoms in the PPMI cohort were recently published and are not included in this article.^11^, ^12^

**Table 3:** Clinical characteristics and DaTSCAN over time in PD subjects

Variable	Baseline	Month 6	Month 12	Month 24	Month 36	Month 48	Month 60	*P* value, change over time
MOCA								<.0001
n completed	420	N/A	392	374	363	339	217	
Mean (SD)	27.13 (2.3)	N/A	26.30 (2.8)	26.27 (3.2)	26.40 (3.0)	26.42 (3.6)	26.42 (3.8)	
(Min, max)	(17.0, 30.0)	N/A	(15.0, 30.0)	(9.0, 30.0)	(13.0, 30.0)	(11.0, 30.0)	(2.0, 30.0)	
MDS‐UPDRS part I								<.0001
n completed	422	403	395	377	366	340	221	
Mean (SD)	5.57 (4.1)	6.40 (4.7)	6.77 (4.6)	7.66 (5.0)	8.32 (5.4)	9.06 (5.9)	9.80 (6.5)	
(Min, max)	(0.0, 24.0)	(0.0, 33.0)	(0.0, 29.0)	(0.0, 26.0)	(0.0, 36.0)	(0.0, 36.0)	(0.0, 36.0)	
MDS‐UPDRS part II								<.0001
n completed	422	403	395	377	366	343	221	
Mean (SD)	5.90 (4.2)	7.81 (5.3)	7.53 (5.1)	7.98 (5.3)	8.91 (5.7)	9.82 (6.7)	10.83 (7.3)	
(Min, max)	(0.0, 22.0)	(0.0, 28.0)	(0.0, 36.0)	(0.0, 27.0)	(0.0, 29.0)	(0.0, 37.0)	(0.0, 40.0)	
Modified Schwab & England ADL								<.0001
n completed	423	401	393	376	365	342	221	
Mean (SD)	93.14 (5.9)	90.92 (7.7)	90.46 (6.7)	88.78 (8.0)	87.66 (8.1)	85.73 (10.4)	83.28 (14.6)	
(Min, max)	(70.0, 100.0)	(50.0, 100.0)	(70.0, 100.0)	(60.0, 100.0)	(50.0, 100.0)	(20.0, 100.0)	(10.0, 100.0)	
Hoehn & Yahr, n (%)^a^								<.0001
Stage 0	0 (0.00)	N/A	1 (0.30)	2 (0.71)	0 (0.00)	1 (0.40)	0 (0.00)	
Stage 1	185 (43.74)	N/A	99 (29.73)	71 (25.09)	45 (17.65)	38 (15.26)	11 (6.75)	
Stage 2	236 (55.79)	N/A	220 (66.07)	197 (69.61)	187 (73.33)	183 (73.49)	141 (86.50)	
Stage 3‐5	2 (0.47)	N/A	13 (3.90)	13 (4.59)	23 (9.02)	27 (10.84)	11 (6.75)	
Contralateral putamen								<.0001
n completed	419	N/A	369	345	N/A	235	3	
Mean (SD)	0.69 (0.3)	N/A	0.60 (0.2)	0.57 (0.2)	N/A	0.49 (0.2)	0.30 (0.2)	
(Min, max)	(0.1, 2.2)	N/A	(0.1, 1.9)	(0.0, 1.6)	N/A	(0.1, 1.6)	(0.1, 0.5)	
Mean putamen								<.0001
n completed	419	N/A	369	345	N/A	235	3	
Mean (SD)	0.83 (0.3)	N/A	0.69 (0.3)	0.65 (0.3)	N/A	0.55 (0.2)	0.48 (0.1)	
(Min, max)	(0.2, 2.2)	N/A	(0.1, 2.3)	(0.0, 1.9)	N/A	(0.1, 1.5)	(0.4, 0.6)	
Mean caudate								<.0001
n completed	419	N/A	369	345	N/A	235	3	
Mean (SD)	2.00 (0.6)	N/A	1.78 (0.5)	1.67 (0.5)	N/A	1.48 (0.5)	1.15 (0.2)	
(Min, max)	(0.4, 3.7)	N/A	(0.3, 3.7)	(0.2, 3.6)	N/A	(0.2, 3.0)	(0.9, 1.4)	
Mean striatum								<.0001
n completed	419	N/A	369	345	N/A	235	3	
Mean (SD)	1.41 (0.4)	N/A	1.24 (0.4)	1.16 (0.4)	N/A	1.01 (0.4)	0.81 (0.1)	
(Min, max)	(0.3, 2.6)	N/A	(0.2, 2.7)	(0.1, 2.4)	N/A	(0.1, 2.0)	(0.7, 1.0)	

Report generated on data submitted as of October 23, 2017. DaTSCAN is not completed at month 36. Contralateral putamen is labeled in regard to the more clinically affected PD body side.

a Hoehn & Yahr *P* value comes from a logistic model comparing Stages 0 to 1 vs 2 to 5.

ADLs, activities of daily living; N/A, not applicable; DAT, Dopamine transporter; DatScan, 123‐I Ioflupane; SPECT, single photon emission computed tomography imaging.

The longitudinal change in DAT binding is detailed in Table 3 and depicted in Figure 1D. There was a significant change in all regions over time. The mean percent reduction (standard deviation) compared to baseline in mean striatum was 11.2 (15.1)/17.0 (16.6)//27.4 (17.3), mean caudate was 9.6 (16.1)/15.7 (16.8)/25.6 (18.3), and mean putamen binding was 13.5 (21.8)/19.1 (21.0)/30.6 (21.3) at years 1, 2, and 4, respectively (Supporting Information Table 7s). The change was greater in the ipsilateral putamen when compared with the contralateral putamen and was greater in year 1 than in subsequent years. Correlation analysis of the regional DAT binding and MDS‐UPDRS at each time point (ie, baseline and years 1, 2, and 4) demonstrated a significant, but small, correlation between MDS‐UPDRS and DAT binding variables most marked at baseline (Table 4). Correlations at year 4 are less significant, but there is a smaller sample size at year 4. The magnitude of correlation increased slightly specifically at year 2 when we reran the correlation analysis in the subset of the participants treated only with levodopa (Table 4). Correlation of the percent change from baseline in regional DAT binding and the change in MDS‐UPDRS showed no significant correlation at either years 1 or 2 (Table 4). At year 4, there was a significant, but small, correlation between change in MDS‐UDPRS total score and percent change in both mean caudate and mean striatum binding, but not mean or contralateral putamen.

**Table 4:** Correlations between change in MDS‐UPDRS and percentage change in DaTSCAN SBR

	Change at year 1		Change at year 2		Change at year 4	
Variable	Spearman correlation coefficient	*P* value	Spearman correlation coefficient	*P* value	Spearman correlation coefficient	*P* value
Correlation with % contralateral putamen						
MDS‐UPDRS part III score	.0800	.1580	−.0117	.8509	−.0501	.5149
MDS‐UPDRS total score	.0256	.6515	−.0708	.2543	−.0739	.3366
Correlation with % mean putamen						
MDS‐UPDRS part III score	.0027	.9624	.0187	.7629	−.0016	.9836
MDS‐UPDRS total score	−.0322	.5701	−.0359	.5641	−.0757	.3248
Correlation with % mean caudate						
MDS‐UPDRS part III score	−.0403	.4775	.0470	.4492	−.1036	.1777
MDS‐UPDRS total score	−.0474	.4030	−.0791	.2025	−.1819	.0173
Correlation with % mean striatum						
MDS‐UPDRS part III score	−.0254	.6548	.0376	.5443	−.0800	.2984
MDS‐UPDRS total score	−.0414	.4654	−.0777	.2107	−.1614	.0349
SBR, specific binding ratio.						

**TABLE 4B.** Correlations between MDS‐UPDRS and DaTSCAN SBR (all patients)								
Variable	Baseline		Year 1		Year 2		Year 4	
	Spearman correlation coefficient	*P* value	Spearman correlation coefficient	*P* value	Spearman correlation coefficient	*P* value	Spearman correlation coefficient	*P* value
Correlation with contralateral putamen								
MDS‐UPDRS part III score	−.2119	<.0001	−.1918	.0006	−.2333	.0001	−.1317	.0860
MDS‐UPDRS total score	−.2100	<.0001	−.2361	<.0001	−.3282	<.0001	−.1854	.0152
Correlation with mean putamen								
MDS‐UPDRS part III score	−.2760	<.0001	−.2130	.0001	−.2513	<.0001	−.1722	.0243
MDS‐UPDRS total score	−.2894	<.0001	−.2511	<.0001	−.3398	<.0001	−.2384	.0017
Correlation with mean caudate								
MDS‐UPDRS part III score	−.1709	.0004	−.1210	.0324	−.1664	0.0070	−.1900	.0128
MDS‐UPDRS total score	−.1820	.0002	−.1483	.0086	−.2655	<.0001	−.2356	.0019
Correlation with mean striatum								
MDS‐UPDRS part III score	−.2246	<.0001	−.1561	.0056	−.2001	.0011	−.1926	.0116
MDS‐UPDRS total score	−.2353	<.0001	−.1881	.0008	−.2996	<.0001	−.2446	.0013
SBR, specific binding ratio.								

**TABLE 4C.** Correlations between MDS‐UPDRS and DaTSCAN SBR in PD patients treated with levodopa only							
Variable	Baseline	Year 1		Year 2		Year 4	
	Not applicable	Spearman correlation coefficient	*P* value	Spearman correlation coefficient	*P* value	Spearman correlation coefficient	*P* value
Correlation with contralateral putamen							
MDS‐UPDRS part III score		−.0911	.5814	−.2972	.0096	−.0687	.6180
MDS‐UPDRS total score		−.0649	.6948	−.4093	.0003	−.1942	.1553
Correlation with mean putamen							
MDS‐UPDRS part III score		−.1464	.3739	−.2437	.0351	−.0328	.8123
MDS‐UPDRS total score		−.1299	.4308	−.3193	.0052	−.1818	.1839
Correlation with mean caudate							
MDS‐UPDRS part III score		−.2309	.1574	−.1886	.1051	−.0948	.4911
MDS‐UPDRS total score		−.1735	.2907	−.3136	.0061	−.2167	.1121
Correlation with mean striatum							
MDS‐UPDRS part III score		−.2230	.1724	−.2027	.0811	−.0857	.5341
MDS‐UPDRS total score		−.1731	.2919	−.3228	.0047	−.2300	.0911

Report generated on data submitted as of October 23, 2017. SBR, specific binding ratio. DAT, DaTSCAN‐Dopamine transporter; DatScan®, 123‐I Ioflupane; SPECT, single photon emission computed tomography imaging.

## Discussion

The PPMI study was designed to accelerate development of therapies for PD by clarifying the performance of clinical and biological markers of disease. In this report, we systematically explore longitudinal change of the MDS‐UPDRS (considering the impact of introducing symptomatic treatment), other clinical measures, and DAT binding in the PPMI cohort. Furthermore, our study provides data on the correlation between the motor clinical outcomes and DAT binding. These data are highly valuable for the design of future disease modification trials.

We conducted an in‐depth analysis of the change of MDS‐UPDRS over year 1 by treatment status with the rationale that these data are frequently used for the design of the disease modification trials in early PD. A number of previously reported studies have provided data on the longitudinal change of UPDRS total score in early at baseline untreated PD cohorts.^13^, ^14^, ^15^, ^16^, ^17^ The change in UPDRS ranges between 6 and 12 points over 1 year. Taking into consideration the UPDRS to MDS‐UPDRS conversion factor of 1.4, our data are consistent with these previously completed studies.^18^ Not surprisingly, our data demonstrate significant differences in the rate of change in the MDS‐UPDRS in participants who initiated DT (42% of the cohort) versus those who remained untreated at 1 year of follow‐up (41%). DT provides a robust symptomatic benefit in early PD, and once DT is initiated, the rate of change of motor disability flattens until participants reach more advanced stages of PD dominated by levodopa‐resistant symptoms.^19^ These data are crucial to design of clinical trials that plan to recruit early untreated PD participants who will require DT even within the first year of evaluation. Interestingly, 17% of the participants who were treated with non‐DT PD medications (MAO‐Bs and/or anticholinergics and amantadine) at year 1 had a change in the MDS‐UPDRS in between the values seen in DT‐treated and untreated individuals, but closer to those who remained untreated. These data reflect the lower potency of these agents. Our data cannot be directly compared to the longitudinal studies that tested efficacy of rasagiline in a de novo population because we assessed cumulative effect of these agents in our analysis.^20^ The PPMI cohort data are in accord with the majority of previously reported studies demonstrating 60% rate of initiation of any PD medication by year 1 (Fig. 1D).^10^ As expected, non‐DT therapies are initiated earlier than major classes of DT.

It is also not surprising that the difference between MDS‐UPDRS OFF and ON scores in the treated participants was very modest given their early stage of disease. That was true even in the subset of the participants treated only with levodopa. Many participants were treated with long‐acting DT agents, such as long‐acting dopamine agonists, and wearing off of symptomatic benefit would be expected to be minimal. In addition, as was demonstrated in other studies,^21^ even in participants treated with levodopa alone, there is a well‐established phenomenon of levodopa long‐duration response with time to wash out of symptomatic benefit exceeding 2 weeks, particularly early in treatment. Although the minimum requirement for OFF time in the PPMI study is 6 hours compared to the 12 hours practically defined OFF state,^22^ the average time to OFF assessment at all time points was >12 hours, and 80% or more of OFF exams were completed after 12 hours, and as such we do not believe it had an impact on the magnitude of OFF/ON difference. Such small delta in OFF/ON scores raises the question of validity of OFF assessments in the early‐PD population, and it might be reasonable to consider ON assessments as a longitudinal outcome in early PD.

Those participants who did not require initiation of DT had milder disease at baseline. At first glance, paradoxically they had a larger and less variable change in MDS‐UPDRS during the 12 and 24 months, but that can be explained by the fact that they do not experience the benefit of robust improvement with DT. Overall, these MDS‐UPDRS data analyzed by DT treatment status provide a scaffold for planning the scope and duration of clinical trials with different sets of assumptions and study inclusion criteria.

We also report the longitudinal change in DAT binding in this cohort. A reduction in DAT binding was an eligibility requirement for the PD participants. During the 4‐year assessment interval, there was a marked additional reduction in DAT binding in all regions. The reduction was evident in all regions, but more marked in the putamen, consistent with the prior studies.^16^ The change in ipsilateral putamen was greater than the change in contralateral putamen at all time points, suggesting that there may be a floor effect limiting the already reduced contralateral putamen. These data also demonstrate that the annualized change in DAT binding was greatest at year 1 when compared with years 2 and 4. These data may be consistent with recent pathology data suggesting that DAT terminal have largely disappeared by year 4 of diagnosis,^23^ again creating a floor effect for change in DAT binding. These data also suggest the limitations of the linear change analysis for DAT binding.

MDS‐UPDRS data and DAT‐binding data show significant but modest correlation at baseline and at years 1, 2, and 4 of evaluation. The modest correlation is explained by the fact that these outcomes measure overlapping but different aspects of PD pathology and are manifest at different stages of the neurodegeneration in PD. Considering that the earliest clinical motor manifestations of PD occur at the point of at least 50% loss of dopaminergic transporter binding, such weak correlation is not surprising. Finally, the comparison of MDS‐UPDRS and DAT binding in years 1 to 4 is confounded by the profound treatment effect of PD medications on motor MDS‐UPDRS scores, although the analysis was run for the OFF scores. The lack of robust correlation between the change in MDS‐UPDRS and percent change in DAT binding is similarly explained by the confounding effect of DT on MDS‐UPDRS change. Correlation of change is further limited because the change in both MDS‐UPDRS and DAT binding is small and variable just as typical clinical progression is slow and subject specific. Given the lack of correlation, DAT binding cannot be considered a surrogate outcome for MDS‐UPDRS in early PD clinical trials. However, despite the lack of correlation with UPDRS, the effect of medications designed to slow disease progression on the longitudinal change in DAT binding may be a valuable tool to assess drug mechanism, particularly in early decision‐making trials.

Some limitations of the PPMI study design have to be acknowledged. The PPMI recruited participants with very early PD who were younger and had less baseline disability than the general PD population, and as such the PPMI cohort cannot be considered and was never intended to be representative of the natural history of PD progression. The primary objective of the PPMI study is to facilitate the development of biomarkers of PD progression, and novel PD therapeutics and demographics of the PPMI cohort are similar to the participants recruited in PD de novo interventional studies. For the same reason, the pattern of PD medications utilization in PPMI cohort is not reflective of the PD population at large. However, interestingly as early as year 2, close to 50% of the treated participants were using levodopa, and by 5 years this figure increased to 83%. Conversely, the percent of participants treated with dopamine agonists remained fairly stable at about 40%. These numbers largely reflect a shift in the prescribing patterns from dopamine agonists to levodopa that occurred during the time that our data were collected. Another limitation is the incomplete dataset on MDS‐UPDRS assessments in the OFF state. As the PPMI is an ongoing study, we are working to increase the OFF data collection to be available at even later time points.

In conclusion, we provide data on the 2 anchor outcomes in the PPMI study: longitudinal change of the MDS‐UPDRS and DAT binding in the cohort of recently diagnosed PD patients. Additional longitudinal clinical, biomarker, and genetic assessments of the PPMI cohort are reported and will be reported in other articles. Our data provide comprehensive information on these measures as participant's progress over time and begin PD treatments. Our results provide a framework for designing studies that incorporate clinical and DAT imaging measures in de novo PD participants. Such studies may signal a more accurate and efficient process toward the development of disease‐modifying treatments for PD.

## Author Roles

1) Research project: A. Conception, B. Organization, C. Execution; 2) Statistical Analysis: A. Design, B. Execution, C. Review and Critique; 3) Manuscript: A. Writing of the first draft, B. Review and Critique.

T.S.: 3A, 3B

A.S.: 2A, 2B, 3A

S.L.: 2A, 2C, 3A, 3B

C.S.C.: 2A, 2C

C.C.G.: 2A, 2C, 3A, 3B

D.J.: 2A, 2B, 2C, 3A, 3B

C.M.T.: 2A, 2B, 2C, 3A, 3B

J.Q.T.: 2A, 2B, 2C, 3A, 3B

L.M.S.: 2A, 2B, 2C, 3A, 3B

J.S.: 2A, 2B, 2C, 3A, 3B

N.S.: 2A, 2B, 2C, 3A, 3B

A.S.: 2A, 2B, 2C, 3A, 3B

K.K.: 2A, 2B, 2C, 3A, 3B

A.W.T.: 2A, 2B, 2C, 3A, 3B

B.M.: 2A, 2B, 2C, 3A, 3B

D.G.: 2A, 2B, 2C, 3A, 3B

L.M.C.: 2A, 2B, 2C, 3A, 3B

D.W.: 2A, 2B, 2C, 3A, 3B

T.F.: 2A, 2B, 2C, 3A, 3B

D.T.T.: 2A, 2B, 2C, 3A, 3B

K.P.: 2A, 2B, 2C, 3A, 3B

V.A.: 2A, 2B, 2C, 3A, 3B

M.F.: 2A, 2B, 2C, 3A, 3B

T.S.: 2A, 2B, 2C, 3A, 3B

S.C.: 2A, 2B, 2C, 3A, 3B

K.M.: 2A, 2B, 2C, 3A, 3B

## Financial disclosures of all authors (for the preceding 12 months)

Tanya Simuni has served as a consultant and received consulting fees from Acadia, Abbvie, Allergan, Anavex, Avid, GE Medical, Eli Lilly and Company, Harbor, Ibsen, IMPAX, Lundbeck, Merz, Inc., the National Parkinson Foundation, Navidea, Pfizer, TEVA Pharmaceuticals, UCB Pharma, Voyager, US World Meds, and the Michael J. Fox Foundation for Parkinson's Research; Dr. Simuni has served as a speaker and received an honorarium from Acadia, IMPAX, Lundbeck, TEVA Pharmaceuticals, and UCB Pharma; Dr Simuni is on the Scientific advisory board for Anavex, Sanofi, Michael J Fox Foundation for Parkinson's Research (MJFF). Dr. Simuni sits on the Advisory Board for IMPAX; Dr. Simuni has received research funding from the National Institute of Neurological Disorders and Stroke (NINDS), MJFF, National Parkinson Foundation (NPF), TEVA Pharmaceuticals, Auspex, Biotie, Civitas, Acorda, Lundbeck, Neuroderm, NINDS, National Institutes of Health (NIH), Northwestern Foundation, and the Michael J. Fox Foundation for Parkinson's Research; Dr. Simuni received funding support for educational programs from GE Medical, TEVA, and Lundbeck. Andrew Siderowf has been an employee of Avid Radiopharmaceuticals, a wholly owned subsidiary of Eli Lilly and Company in the past 12 months. Shirley Lasch is employed by Molecular NeuroImaging, LLC. Christopher S. Coffey served as a consultant receiving consulting fees from The Michael J. Fox Foundation for Parkinson's Research; received research funding from NINDS, NHLBI, and The Michael J. Fox Foundation for Parkinson's Research. Chelsea Caspell‐Garcia **s**erved as a consultant receiving consulting fees from The Michael J. Fox Foundation for Parkinson's Research; Received research funding from The Michael J. Fox Foundation for Parkinson's Research. Danna Jennings is an employee of Eli Lilly. Caroline M. Tanner is an employee of the San Francisco Veterans Affairs Medical Center and the University of California – San Francisco. She receives grants from the Michael J. Fox Foundation, the Parkinson's Disease Foundation, the Department of Defense, Sage Bionetworks and the National Institutes of Health, compensation for serving on Data Monitoring Committees from Biotie Therapeutics, Voyager Therapeutics and Intec Pharma and personal fees for consulting from Neurocrine Biosciences, Adamas Pharmaceuticals, Photopharmics, and 23andMe. Daniel Weintraub has received research funding or support from Michael J. Fox Foundation for Parkinson's Research, National Institutes of Health (NINDS), Novartis Pharmaceuticals, Department of Veterans Affairs, Avid Radiopharmaceuticals, Alzheimer's Disease Cooperative Study, and the International Parkinson and Movement Disorder Society; honoraria for consultancy from Acadia, Biogen, Biotie (Acorda), Bracket, Clintrex LLC, Eisai Inc., Eli Lilly, Lundbeck, Roche, Takeda, UCB, and the CHDI Foundation; license fee payments from the University of Pennsylvania for the Questionnaire for Impulsive‐Compulsive Disorders in Parkinson's Disease (QUIP) and Questionnaire for Impulsive‐Compulsive Disorders in Parkinson's Disease ‐ Rating Scale (QUIP‐RS); royalties from Wolters Kluweland; and fees for legal consultation for lawsuits related to medication prescribing in patients with Parkinson's disease. Lana M. Chahine receives support from the Michael J Fox Foundation and receives royalties from Wolters Kluwel (for book authorship). John Trojanowski may accrue revenue in the future on patents submitted by the University of Pennsylvania wherein he is co‐Inventor and he received revenue from the sale of Avid to Eli Lily as co‐inventor on imaging related patents submitted by the University of Pennsylvania. Leslie M. Shaw has received consulting fees from Roche, Lilly and Norvatis, has served on scientific advisory boards for Roche, Lilly and Norvatis, receives support from the NIA, The Michael J. Fox Foundation for Parkinson's Research, Roche, and Lilly. Karl Kieburtz has served as a consultant and received consulting fees from: Acorda, Astellas Pharma, AstraZeneca, BioMarin Pharmaceutica, Biotie, Britannia, CHDI, Clearpoint Strategy Group, Clintrex, Corium International, Cynapsus, Forward Pharma, Genzyme, INC Research, Intec, Lundbeck, Medivation, Melior Discovery, Neurocrine, Neuroderm, Neurmedix, Orion Pharma, Otsuka, Pfizer, Pharma2B, Prana Biotechnology, Prothena/Neotope/Elan Pharmaceutical, Raptor Pharmaceuticals, Remedy Pharmaceuticals, Roche/Genentech, Sage Bionetworks, Sanofi, Serina, Sunovion, Synagile, Titan, Upsher‐Smith, US WorldMeds, Vaccinex, Vertex Pharmaceuticals, Voyager, and Weston Brain Institute. Dr. Kieburtz has received funding from National Institutes of Health (NINDS), The Michael J Fox Foundation, and Teva. Kathleen L. Poston receives funding from The Michael J. Fox Foundation and the National Institutes of Health. Thomas Comery is employed by Pfizer, Inc. Brit Mollenhauer is employed by Parcacelsus Kliniken Germany and the University Medical Center Goettingen; BM has received independent research grants from TEVA‐Pharma, Desitin, Boehringer Ingelheim, GE Healthcare and honoraria for consultancy from Bayer Schering Pharma AG, Roche, AbbVie, TEVA‐Pharma, Biogen and for presentations from GlaxoSmithKline, Orion Pharma, TEVA‐Pharma and travel costs from TEVA‐Pharma. BM is member of the executive steering committee of the Parkinson Progression Marker Initiative and the Systemic Synuclein Sampling Study of the Michael J. Fox Foundation for Parkinson's Research and has received grants from the Bundesministerium fur Bildung und Forschung (BMBF), European Union (EU), Parkinson Fonds Deutschland, Deutsche Parkinson Vereinigung, Michael J. Fox Foundation for Parkinson's Research, Stifterverband für die deutsche Wissenschaft, and has scientific collaborations with Roche, Bristol Myers Squibb, Ely Lilly, Covance and Biogen. Douglas Galasko receives research funding from National Institutes of Health (NIH), Michael J. Fox Foundation, and Eli Lilly and Esai. He is a paid Editor for Alzheimer's Research and Therapy. He is a consultant for vTv Therapeutics and serves on a DSMB for Prothena. Tatiana Foroud receives funding from the National Institutes of Health, The Michael J. Fox Foundation, the U.S. Department of Defense. Dr. Foroud has received funding from The Michael J. Fox Foundation, the NIH, San Diego State University, The University of Texas at Austin, and Waggoner Center for Alcohol/Addiction Research. Vanessa Arnedo is employed by The Michael J. Fox Foundation. Mark Frasier is employed by The Michael J. Fox Foundation. Todd Sherer is employed by The Michael J. Fox Foundation. Sohini Chowdhury is employed by The Michael J. Fox Foundation. Kenneth Marek receives funding from the The Michael J. Fox Foundation, the US Department of Defense and is employed by Invicro and has received consultant fees from. Pfizer, GE Healthcare, Lilly, BMS, Piramal, Biogen, Prothena, Roche, Neuropore, US Worldmeds, Neurophage, UCB, Oxford Biomedica, Lysosomal Therapetic, Inc, Neuroderm, and Denali.

John Siebyl served as a consultant receiving consulting fees from GE Healthcare, Piramal Imaging, Roche, and Biogen. Dr. Siebyl has received research funding from MJFF.

Andrew Singleton was supported in part by the Intramural Research Program of the National Institute on Aging, National Institutes of Health, Department of Health and Human Services; project ZO1 AG000949.

## Supporting information

Additional Supporting Information may be found in the online version of this article at the publisher's website.

Supplementary Information 1

## Parkinson's Progression Marker Initiative Authors

**PPMI Steering Committee**: Kenneth Marek, MD^1^ (Principal Investigator); Shirley Lasch, MBA^1^; Caroline Tanner, MD, PhD^2^ (Site Investigator); Tanya Simuni, MD^3^ (Site Investigator); Christopher Coffey, PhD^4^ (Statistics Core, PI); Karl Kieburtz, MD, MPH^5^ (Clinical Core, PI); Renee Wilson^5^; Brit Mollenhauer, MD^6^ (Bioanalytics Core, co‐PI; Site Investigator); Douglas Galasko, MD^7^ (Bioanalytics Core, co‐PI; Site Investigator); Tatiana Foroud, PhD^8^ (Genetics Coordination Core and Biorepository, PI); Lana Chahine, MD^9^ (Site Investigator); Andrew Siderowf, MD, MSCE^9;^ John Seibyl, MD (Imaging Core, PI)^1^; Arthur Toga, PhD^10^ (Bioinformatics Core, PI); Andrew Singleton, PhD^11^ (Genetics Core, PI); Daniel Weintraub, MD^9^ (Cognitive and Behavioral); John Trojanowski, MD, PhD^9^; Leslie Shaw, PhD^9^; Duygu Tosun‐Turgut, PhD^2^ (DTI, PI); Kathleen Poston, MD, MS (fMRI, PI)^15^; Susan Bressman, MD^27^; Kalpana M. Merchant, MD^54^; Werner Poewe, MD^12^ (Site Investigator); Todd Sherer, PhD^13^; Sohini Chowdhury^13^; Mark Frasier, PhD^13^; Catherine Kopil, PhD^13^; Anna Naito, PhD^13^; Vanessa Arnedo.^13^ **PPMI Study Cores (additional members)**: *Clinical Coordination Core*: Ray Dorsey, PhD^5^; Cynthia Casaceli, MBA^5^; *Imaging Core*: Nichole Daegele^1^; Justin Albani^1^ *Statistics Core*: Chelsea Caspell‐Garcia, MS ^4^; Liz Uribe, MS^4^; Eric Foster ^4^; Jeff Long, PhD^4^; Nick Seedorff^4^; *Bioinformatics Core*: Karen Crawford, MLIS^10^; *BioRepository*: Danielle Elise Smith^8^; Paola Casalin^14^; Giulia Malferrari^14^; *Genetics Coordination and Pathology Core*: Cheryl Halter ^8^; Laura Heathers.^8^ **PPMI Site Investigators**: David Russell, MD, PhD^1^; Stewart Factor, DO^16^; Penelope Hogarth, MD^17^; David Standaert, MD, PhD^18^; Amy Amara, MD, PhD^18^; Robert Hauser, MD, MBA^19^; Joseph Jankovic, MD^20^; Matthew Stern, MD^9^; Shu‐Ching Hu, MD PhD^21^; Gretchen Todd^21^; Rachel Saunders‐Pullman MD^27^; Irene Richard, MD^23^; Marie H Saint‐Hilaire, MD^22^; Klaus Seppi, MD^12^; Holly Shill, MD^24^; Hubert Fernandez, MD^25^; Claudia Trenkwalder, MD^6^; Wolfgang Oertel MD^42^ ; Daniela Berg, MD^26^; Kathrin Brockman, MD^26^; Isabel Wurster MD^26^; Liana Rosenthal, MD^28^; Yen Tai, MD^29^; Nicola Pavese, MD^29^; Paolo Barone, MD, PhD^30^; Stuart Isaacson, MD^31^; Alberto Espay, MD, MSc^32^; Dominic Rowe, MD, PhD^33^; Melanie Brandabur MD^35^; James Tetrud MD^35^; Grace Liang MD^35^; Alex Iranzo, MD^34^; Eduardo Tolosa MD^34^; Karen Marder, MD^36^; Maria de Arriba Sanchez, MD^37^; Leonidis Stefanis, MD, PhD^38^; Maria Jose Marti, MD, PhD^34^; Javier Ruiz Martinez, MD, PhD^37^; Jean‐Christophe Corvol, MD^39^; Jan O Assly, MD^40^; Salima Brillman, MD^35^; Nir Giladi, MD.^41^ **PPMI Coordinators**: Debra Smejdir^1^; Julia Pelaggi ^1^; Farah Kausar, PhD^2^; Linda Rees, MPH^35^; Barbara Sommerfield, MSN, RN^16^; Madeline Cresswell^17^; Courtney Blair, MA^18^; Karen Williams^3^; Grace Zimmerman^5^; Stephanie Guthrie, MSN^18^; Ashlee Rawlins^18^; Leigh Donharl^19^; Christine Hunter, RN^20^; Baochan Tran^9^; Abigail Darin^9^; Carly Linder^9^; Marne Baca^21^; Heli Venkov^21^; Cathi‐Ann Thomas, RN, MS^22^; Raymond James, RN^22^; Beatrice Heim, MD^12^; Paul Deritis^23^; Fabienne Sprenger, MD^12^; Deborah Raymond^27^; Diana Willeke^6^; Zoran Obradov, CRC^24^; Jennifer Mule^25^; Nancy Monahan^25^; Katharina Gauss^26^; Deborah Fontaine, BSN, MS^7^; Daniel Szpak^7^; Arita McCoy^28^; Becky Dunlop^28^; Laura Marie Payne^29^; Susan Ainscough^30^; Lisbeth Carvajal^31^; Rebecca Silverstein^31^; Kristy Espay^32^; Madelaine Ranola^33^; Elisabet Mondragon Rezola^37^; Helen Mejia Santana^36^; Maria Stamelou, MD, PhD^38^; Alicia Garrido, MD^34^; Stephanie Carvalho, MS^39^; Anne Grete Kristiansen^40^; Krista Specketer^21^ ; Anat Mirlman.^41^ **ISAB (Industry Scientific Advisory Board)**: Maurizio Facheris, MD^43^; Holly Soares, PhD^43^; Mark A. Mintun, MD^44^; Jesse Cedarbaum, MD^45^; Peggy Taylor, ScD^46^; Danna Jennings, MD^48^; Lawrence Slieker, PhD^48^; Brian McBride, PhD^49^; Colin Watson, PhD^49^; Etienne Montagut, MBA^49^; Zulfiqar Haider Sheikh^49^; Baris Bingol, PhD^50^; Remi Forrat^51^; Pablo Sardi, PhD^51^; Tanya Fischer, MD, PhD^51^; Alastair D. Reith, PhD^52^; Jan Egebjerg, PhD^53^; Lone Frydelund Larsen^53^; Nathalie Breysse, PhD^53^; Didier Meulien, MD^53^; Barbara Saba, MD^54^; Vera Kiyasova, MD, PhD^54^; Chris Min, MD, PhD^55^; Thomas McAvoy, PhD^55^; Robert Umek, PhD^56^; Philip Iredale, PhD^57^; Jeremy Edgerton, PhD^57^; Susan De Santi, PhD^58^; Christian Czech, PhD^59^; Frank Boess, PhD^59^; Jeffrey Sevigny, MD^59^; Thomas Kremer, PhD^59^; Igor Grachev, MD, PhD^60^; Kaplana Merchant, PhD^61^; Andreja Avbersek, MD^62^; Pierandrea Muglia, MD^62^; Alexandra Stewart, MBA^63^; Rene Prashad, PhD^63^, Johannes Taucher, MD^64^

1 Institute for Neurodegenerative Disorders, New Haven, CT

2 University of California, San Francisco, CA

3 Northwestern University, Chicago, IL

4 University of Iowa, Iowa City, IA

5 Clinical Trials Coordination Center, University of Rochester, Rochester, NY

6 Paracelsus‐Elena Klinik, Kassel, Germany

7 University of California, San Diego, CA

8 Indiana University, Indianapolis, IN

9 University of Pennsylvania, Philadelphia, PA

10 Laboratory of Neuroimaging (LONI), University of Southern California, Los Angeles, CA

11 National Institute on Aging, NIH, Bethesda, MD

12 Innsbruck Medical University, Innsbruck, Austria

13 The Michael J. Fox Foundation for Parkinson's Research, New York, NY

14 BioRep Milan, Italy

15 Stanford University Medical Center, Stanford, CA

16 Emory University of Medicine, Atlanta, GA

17 Oregon Health and Science University, Portland, OR

18 University of Alabama at Birmingham, Birmingham, AL

19 University of South Florida, Tampa, FL

20 Baylor College of Medicine, Houston, TX

21 University of Washington/ University of Washington and VA Puget Sound Health, Seattle, WA

22 Boston University, Boston, MA

23 University of Rochester, Rochester, NY

24 Banner Research Institute, Sun City, AZ

25 Cleveland Clinic, Cleveland, OH

26 University of Tuebingen, Tuebingen, Germany

27 Beth Israel Medical Center, New York, NY

28 Johns Hopkins University, Baltimore, MD

29 Imperial College of London, London, UK

30 University of Salerno, Salerno, Italy

31 Parkinson's Disease and Movement Disorders Center, Boca Raton, FL

32 University of Cincinnati, Cincinnati, OH

33 Macquarie University, Sydney Australia

34 Hospital Clinic of Barcelona, Barcelona, Spain

35 The Parkinson's Institute, Sunnyvale, CA

36 Columbia University Medical Center, New York, NY

37 Hospital Donista, San Sebastian, Spain

38 Foundation for Biomedical Research of the Academy of Athens, Athens, Greece

39 Hospital Pitie‐Salpetriere, Paris France

40 St Olav's Hospital, Norway

41 Tel Aviv Sourasky Medical Center, Tel Aviv, Israel

42 Philipps University Marburg, Germany

44 Avid Radiopharmaceuticals, Inc, Philadelphia, PA

48 Eli Lilly and Company, New York, NY

43 Abbvie, Chicago, IL

45 Biogen Idec, Cambridge, MA

46 BioLegend, San Diego, CA

47 Bristol‐Myers Squibb Company, New York, NY

49 GE Healthcare, Little Chalfont, United Kingdom

50 Genentech Inc., South San Francisco, CA

51 Genyzme Sanofi, Cambridge, MA

52 GlaxoSmithKline Pharmaceuticals R&D, Brentford, United Kingdom

53 H. Lundbeck A/S Copenhagen, Denmark

54 Institut de Recherches Internationales Servier, Croissy, France

55 Merck, Kenilworth, NJ

56 Meso Scale Discovery Rockville, MD

57 Pfizer Inc, Cambridge, MA

58 Piramal Life Sciences, Berlin, Germany

59 Roche, Basel, Switzerland

60 Teva, Petah Tekva, Israel

61 TransThera Consulting Co., Portland, OR

62 UCB Pharma S.A., Brussels, Belgium

63 Weston Brain Institute, Toronto, ON

64 Takeda, Osaka, Japan
