# Supplementary material for: Longitudinal Change of Clinical and Biological Measures in Early Parkinson's Disease: Parkinson's Progression Markers Initiative Cohort
Source: Mov Disord. 2018 Mar 23;33(5):771–82. doi: 10.1002/mds.27361 (PMC6001458; doi:10.1002/mds.27361)
Supplement: Supplementary file 1 — Supplementary Information 1 [file MDS-33-771-s001.docx]

**Supplementary Tables**

**Table 1s. Change in MDS-UPDRS Total Scores from BL to Year 1 in PD Subjects**

|  | **Subjects that completed BL and Year 1 Visits** | | | | **p-value** | | |
| --- | --- | --- | --- | --- | --- | --- | --- |
| **Variable** | **All PD** | **Untreated** | **Treated with** | **Treated with** | **Untreated vs.** | **Untreated vs.** | **Levodopa/DA** |
|  | **Subjects** | **at Year 1** | **Levodopa/DA at Year 1** | **Other PD Med at Year 1** | **Levodopa/DA** | **Other** | **vs. Other** |
|  | (N = 393) | (N = 162) | (N = 165) | (N = 66) |  |  |  |
| **Change in MDS-UPDRS Total Score** |  |  |  |  | <.0001 | 0.0406 | 0.0047 |
| N Completed | 334 | 162 | 106 | 66 |  |  |  |
| Mean (SD) | 7.45 (11.6) | 10.73 (10.7) | 2.41 (11.4) | 7.48 (11.2) |  |  |  |
| (Min, Max) | (-31.0, 60.0) | (-17.0, 60.0) | (-31.0, 32.0) | (-19.0, 41.0) |  |  |  |

**Table 2s. Time between Dose and MDS-UPDRS OFF Score by Visit in Treated PD Subjects**

|  | **Month 12** | **Month 24** | **Month 36** | **Month 48** | **Month 60** |
| --- | --- | --- | --- | --- | --- |
|  | (N = 106) | (N = 163) | (N = 186) | (N = 210) | (N = 146) |
| **Time to OFF Evaluation** |  | | | | |
| Mean (SD) | 16.72 (6.2) | 16.32 (5.6) | 15.53 (5.4) | 14.31 (4.4) | 14.11 (4.5) |
| (Min, Max) | (6.0, 32.5) | (6.0, 36.0) | (6.0, 47.0) | (6.0, 36.0) | (6.0, 38.0) |
| Missing | 0 | 0 | 0 | 0 | 1 |
| **Time to OFF Evaluation** |  | | | | |
| 6-9 hours | 11 (10.38%) | 9 (5.52%) | 14 (7.53%) | 15 (7.14%) | 8 (5.48%) |
| 9-12 hours | 14 (13.21%) | 19 (11.66%) | 26 (13.98%) | 36 (17.14%) | 31 (21.23%) |
| >12 hours | 81 (76.42%) | 135 (82.82%) | 146 (78.49%) | 159 (75.71%) | 106 (72.60%) |

*Note: Time to evaluation was not collected at the 6 Month visit.

**Table 3s. Year 1 Change in MDS-UPDRS Total Scores by Time to OFF Evaluation in Treated PD Subjects**

| **Variable** | **Time to OFF Score at Year 1** | |  |
| --- | --- | --- | --- |
|  | **6-12 Hours** | **>12 Hours** | **p-value** |
| **Change in MDS-UPDRS Total Score** |  | | 0.4718 |
| N Completed | 25 | 81 |  |
| Mean (SD) | 0.96 (13.6) | 2.85 (10.7) |  |
| (Min, Max) | (-18.0, 31.0) | (-31.0, 32.0) |  |

*Note: Treatment status for this analysis is defined as treatment with either levodopa or dopamine agonists.

Report Generated on Data Submitted as of: 23Oct2017.

**Table 4s. PD Medications over Time in Treated PD Subjects**

|  | **Treated* PD Subjects** | | | | |
| --- | --- | --- | --- | --- | --- |
| **Variable** | **Month 12** | **Month 24** | **Month 36** | **Month 48** | **Month 60** |
|  | (N = 241) | (N = 322) | (N = 342) | (N = 321) | (N = 209) |
| **Total LED** |  |  |  |  |  |
| N | 212 | 290 | 307 | 294 | 193 |
| Mean (SD) | 288.65 (217.61) | 381.96 (291.83) | 462.29 (321.65) | 542.87 (332.67) | 694.62 (758.25) |
| (Min, Max) | (30.30, 1600.00) | (50.00, 2268.00) | (40.00, 2474.00) | (50.00, 3020.00) | (137.50, 9470.00) |
| **LED Subtotal - Levodopa +/- Entacopone** |  |  |  |  |  |
| N | 78 | 144 | 194 | 221 | 161 |
| Mean (SD) | 405.77 (258.47) | 455.65 (299.60) | 472.68 (316.26) | 491.92 (306.03) | 558.29 (424.53) |
| (Min, Max) | (50.00, 1600.00) | (50.00, 2000.00) | (50.00, 2324.00) | (50.00, 2870.00) | (50.00, 3600.00) |
| **LED Subtotal - Dopamine Agonists** |  |  |  |  |  |
| N | 88 | 125 | 139 | 130 | 83 |
| Mean (SD) | 158.44 (93.04) | 180.41 (127.76) | 185.03 (118.53) | 194.16 (125.37) | 299.00 (982.58) |
| (Min, Max) | (12.50, 450.00) | (7.50, 825.00) | (5.00, 675.00) | (7.50, 675.00) | (37.50, 9090.00) |
| **LED Subtotal - Levodopa or Dopamine Agonists** |  |  |  |  |  |
| N | 157 | 240 | 273 | 272 | 187 |
| Mean (SD) | 290.40 (237.58) | 367.36 (293.91) | 430.11 (322.12) | 492.48 (324.77) | 613.38 (769.39) |
| (Min, Max) | (12.50, 1600.00) | (7.50, 2000.00) | (5.00, 2474.00) | (25.00, 3020.00) | (37.50, 9370.00) |
| **LED Subtotal - other PD meds** |  |  |  |  |  |
| N | 212 | 290 | 307 | 294 | 193 |
| Mean (SD) | 73.58 (84.51) | 77.94 (94.21) | 79.81 (97.15) | 87.24 (111.04) | 100.31 (118.42) |
| (Min, Max) | (0.00, 500.00) | (0.00, 528.00) | (0.00, 500.00) | (0.00, 664.00) | (0.00, 500.00) |
| **Classes of PD Medications**** |  |  |  |  |  |
| Levodopa +/- Entacopone | 85 (35.27%) | 157 (48.76%) | 213 (62.28%) | 237 (73.83%) | 173 (82.78%) |
| MAO-B Inhibitors (+/- other PD meds) | 112 (46.47%) | 150 (46.58%) | 157 (45.91%) | 146 (45.48%) | 92 (44.02%) |
| MAO-B Inhibitors only | 43 (17.84%) | 34 (10.56%) | 20 (5.85%) | 10 (3.12%) | 2 (0.96%) |
| Dopamine Agonists | 94 (39.00%) | 138 (42.86%) | 154 (45.03%) | 142 (44.24%) | 90 (43.06%) |
| Amantadine | 27 (11.20%) | 41 (12.73%) | 49 (14.33%) | 52 (16.20%) | 47 (22.49%) |
| Anticholinergics | 6 (2.49%) | 8 (2.48%) | 15 (4.39%) | 14 (4.36%) | 8 (3.83%) |
| Levodopa + DA | 10 (4.15%) | 34 (10.56%) | 69 (20.18%) | 86 (26.79%) | 64 (30.62%) |
| Any 2 classes besides Levodopa + DA | 69 (28.63%) | 113 (35.09%) | 106 (30.99%) | 97 (30.22%) | 61 (29.19%) |
| Any 3 classes | 6 (2.49%) | 23 (7.14%) | 52 (15.20%) | 65 (20.25%) | 53 (25.36%) |

*Note: This table includes subjects treated with any PD medication.

**Subjects may be taking more than one class of PD medication.

Report Generated on Data Submitted as of: 23Oct2017.

**Table 5s. Number of Expected and Seen PD Subjects by Visit**

| **Subjects** | **Baseline** | **Month 6** | **Month 12** | **Month 24** | **Month 36** | **Month 48** | **Month 60** |
| --- | --- | --- | --- | --- | --- | --- | --- |
| **Expected** | 423 | 414 | 409 | 399 | 388 | 377 | 240 |
| **Seen** |  | | | | | | |
| All Subjects | 423 | 402 | 394 | 376 | 365 | 335 | 218 |
| Untreated | 423 | 373 | 162 | 58 | 27 | 16 | 9 |
| Treated: Levodopa/DA | 0 | 20 | 166 | 257 | 296 | 296 | 201 |
| Treated: Levodopa only | 0 | 11 | 73 | 114 | 124 | 118 | 84 |
| Treated: Other | 0 | 9 | 66 | 61 | 42 | 23 | 8 |

Subjects are expected at the visit if they are past the expected visit window and have not terminated early from the study.

Report Generated on Data Submitted as of: 23Oct2017.

**Table 6s. MDS-UPDRS Changes from BL Over Time in Treated and Untreated PD Subjects**

|  | **Change** | **Change** | **Change** | **Change** | **Change** |
| --- | --- | --- | --- | --- | --- |
|  | **at Year 1** | **at Year 2** | **at Year 3** | **at Year 4** | **at Year 5** |
| **Untreated + Treated OFF** |  |  |  |  |  |
| **Total Score** |  |  |  |  |  |
| N Completed | 334 | 281 | 255 | 249 | 163 |
| Mean (SD) | 7.45 (11.6) | 10.38 (12.9) | 14.31 (15.5) | 19.07 (16.5) | 20.88 (17.7) |
| **Part III Score** |  |  |  |  |  |
| N Completed | 334 | 282 | 255 | 249 | 163 |
| Mean (SD) | 4.51 (8.2) | 6.31 (9.3) | 8.83 (10.9) | 11.57 (11.2) | 12.28 (11.8) |
| **Untreated + Treated ON** |  |  |  |  |  |
| **Total Score** |  |  |  |  |  |
| N Completed | 381 | 353 | 342 | 321 | 204 |
| Mean (SD) | 5.39 (12.7) | 6.96 (13.4) | 9.80 (16.6) | 11.93 (18.0) | 15.19 (19.5) |
| **Part III Score** |  |  |  |  |  |
| N Completed | 382 | 354 | 343 | 321 | 204 |
| Mean (SD) | 2.54 (9.1) | 2.60 (10.1) | 3.65 (11.8) | 4.02 (12.6) | 5.65 (12.4) |
| **Untreated** |  |  |  |  |  |
| **Total Score** |  |  |  |  |  |
| N Completed | 162 | 58 | 27 | 16 | 9 |
| Mean (SD) | 10.73 (10.7) | 14.50 (12.2) | 17.00 (14.1) | 19.56 (22.1) | 10.78 (6.9) |
| **Part III Score** |  |  |  |  |  |
| N Completed | 162 | 58 | 27 | 16 | 9 |
| Mean (SD) | 6.89 (7.0) | 9.81 (8.6) | 12.19 (9.7) | 11.81 (12.0) | 7.22 (6.1) |
| **Levodopa/DA OFF** |  |  |  |  |  |
| **Total Score** |  |  |  |  |  |
| N Completed | 106 | 162 | 186 | 210 | 146 |
| Mean (SD) | 2.41 (11.4) | 8.38 (13.4) | 12.84 (15.9) | 19.05 (16.5) | 21.73 (18.2) |
| **Part III Score** |  |  |  |  |  |
| N Completed | 106 | 163 | 186 | 210 | 146 |
| Mean (SD) | 0.77 (7.9) | 4.58 (9.3) | 7.41 (11.0) | 11.29 (11.4) | 12.65 (12.2) |
| **Levodopa/DA ON** |  |  |  |  |  |
| **Total Score** |  |  |  |  |  |
| N Completed | 153 | 234 | 273 | 282 | 187 |
| Mean (SD) | -1.18 (12.4) | 3.84 (13.1) | 7.66 (16.6) | 10.94 (18.1) | 15.33 (20.1) |
| **Part III Score** |  |  |  |  |  |
| N Completed | 154 | 235 | 274 | 282 | 187 |
| Mean (SD) | -2.93 (8.3) | -0.48 (9.3) | 1.39 (11.1) | 2.77 (12.4) | 5.34 (12.7) |
| **Other PD Meds** |  |  |  |  |  |
| **Total Score** |  |  |  |  |  |
| N Completed | 66 | 61 | 42 | 23 | 8 |
| Mean (SD) | 7.48 (11.2) | 11.77 (11.2) | 19.10 (13.9) | 18.87 (11.0) | 16.75 (12.5) |
| **Part III Score** |  |  |  |  |  |
| N Completed | 66 | 61 | 42 | 23 | 8 |
| Mean (SD) | 4.65 (9.1) | 7.59 (8.7) | 12.93 (9.9) | 13.91 (9.2) | 11.13 (8.3) |

Report Generated on Data Submitted as of: 23Oct2017.

**Table 7s. Percent Change in DaTSCAN Over Time in PD Subjects**

| **Variable** | **% Change**  **at Year 1** | **% Change**  **at Year 2** | **% Change**  **at Year 4** |
| --- | --- | --- | --- |
| **Contralateral Putamen** |  |  |  |
| N Completed | 369 | 345 | 235 |
| Mean (SD) | -7.24 (32.5) | -13.74 (30.5) | -23.96 (27.8) |
| (Min, Max) | (-77.4, 204.8) | (-92.5, 207.1) | (-79.6, 143.8) |
| **Mean Putamen** |  |  |  |
| N Completed | 369 | 345 | 235 |
| Mean (SD) | -13.49 (21.8) | -19.07 (21.4) | -30.61 (21.3) |
| (Min, Max) | (-83.1, 141.2) | (-86.7, 167.7) | (-83.3, 123.2) |
| **Mean Caudate** |  |  |  |
| N Completed | 369 | 345 | 235 |
| Mean (SD) | -9.65 (16.1) | -15.74 (16.8) | -25.63 (18.3) |
| (Min, Max) | (-59.8, 164.9) | (-87.7, 136.7) | (-82.9, 96.0) |
| **Mean Striatum** |  |  |  |
| N Completed | 369 | 345 | 235 |
| Mean (SD) | -11.17 (15.1) | -17.04 (16.6) | -27.43 (17.3) |
| (Min, Max) | (-59.6, 124.8) | (-87.5, 146.2) | (-83.0, 104.3) |

Report Generated on Data Submitted as of: 23Oct2017.

**Table 8s. MDS-UPDRS OFF vs. ON Scores in Treated PD Subjects**

| **Variable** | **Treated with Levodopa/DA** | | | **Treated with Levodopa only** | | |
| --- | --- | --- | --- | --- | --- | --- |
|  | **OFF Score** | **ON Score** | **p-value** | **OFF Score** | **ON Score** | **p-value** |
| **Year 1 MDS-UPDRS Total Score** |  |  | 0.0760 |  |  | 0.1237 |
| N | 106 | 154 |  | 41 | 73 |  |
| Mean (SD) | 39.08 (17.5) | 35.32 (16.1) |  | 43.41 (19.5) | 37.81 (17.9) |  |
| (Min, Max) | (8.0, 89.0) | (4.0, 83.0) |  | (12.0, 89.0) | (6.0, 83.0) |  |
| **Year 1 MDS-UPDRS Part III Score** |  |  | 0.0055 |  |  | 0.0063 |
| N | 106 | 154 |  | 41 | 73 |  |
| Mean (SD) | 24.31 (11.9) | 20.40 (10.5) |  | 27.37 (12.5) | 21.18 (10.7) |  |
| (Min, Max) | (2.0, 60.0) | (1.0, 50.0) |  | (9.0, 60.0) | (2.0, 50.0) |  |
| **Year 2 MDS-UPDRS Total Score** |  |  | 0.0001 |  |  | 0.0050 |
| N | 163 | 235 |  | 81 | 105 |  |
| Mean (SD) | 43.65 (17.5) | 37.05 (16.1) |  | 45.37 (18.9) | 37.77 (17.4) |  |
| (Min, Max) | (10.0, 96.0) | (5.0, 81.0) |  | (10.0, 96.0) | (5.0, 80.0) |  |
| **Year 2 MDS-UPDRS Part III Score** |  |  | <.0001 |  |  | 0.0001 |
| N | 163 | 235 |  | 81 | 105 |  |
| Mean (SD) | 26.91 (11.5) | 20.94 (10.8) |  | 27.83 (11.5) | 20.86 (11.4) |  |
| **(Min, Max)** | (3.0, 62.0) | (0.0, 56.0) |  | (3.0, 59.0) | (0.0, 56.0) |  |

Report Generated on Data Submitted as of: 23Oct2017.
